# Effects of Exposure Parameters and Voxel Size for Cone-Beam Computed Tomography on the Image Matching Accuracy with an Optical Dental Scan Image: An In Vitro Study

**DOI:** 10.1155/2021/6971828

**Published:** 2021-06-10

**Authors:** Hang-Nga Mai, Du-Hyeong Lee

**Affiliations:** ^1^Institute for Translational Research in Dentistry, Kyungpook National University, 2177 Dalgubeoldae-ro, Jung-Gu, Daegu 41940, Republic of Korea; ^2^Department of Prosthodontics, School of Dentistry, Kyungpook National University, 2177 Dalgubeoldae-ro, Jung-Gu, Daegu 41940, Republic of Korea

## Abstract

This study is aimed at assessing the effects of exposure parameters and voxel size for cone-beam computed tomography (CBCT) on the image matching accuracy with an optical dental scan image. CBCT and optical scan images of a dry human mandible were obtained. Different CBCT settings were used: tube voltage, 60, 80, and 100 kVp; tube current, 6 and 8 mA; and voxel size, 100, 200, and 300 *μ*m. Image matching between the CBCT and optical scan images was performed using implant planning software by dental professionals (*n* = 18). The image matching accuracy in each combination of CBCT settings was evaluated by assessing the linear discrepancy between the three-dimensionally reconstructed radiological image and the registered optical scan image using an image analysis software program. The Kruskal-Wallis test and a post hoc Mann–Whitney *U* test with Bonferroni correction were used to compare the accuracy of image registration between the groups (*α* = 0.05). Overall, the image matching accuracy was not significantly different between tube voltage and current settings; however, significantly higher image registration errors were found at the combination of 100 kVp tube voltage/8 mA tube current (*F* = 8.44, *P* < 0.001). Changes in voxel sizes did not significantly interfere with the image registration results. No interaction was found among voltage, current, and voxel size in terms of image registration accuracy (*F* = 2.022, *P* = 0.091). Different exposure parameter settings in tube voltage and tube current did not significantly influence the image matching accuracy between CBCT and optical dental scan images; however, a high radiation dose could be inappropriate. The image matching accuracy was not significantly affected by changing the voxel sizes of CBCT.

## 1. Introduction

Cone-beam computed tomography (CBCT) has become a vital imaging modality in dental therapeutic procedures [1]. Different radiation doses and image quality are needed for various diagnoses, such as periapical pathologic lesions, maxillary sinus diseases, and implant placement [2, 3]. The quality of images obtained by CBCT depends on various exposure parameters, such as tube voltage, exposure duration, tube current, rotation trace, and field of view (FOV) size [4]. Image noise can be decreased as tube voltage and current increase owing to the increase of the detector signal [2]. A higher radiation dose represents a potential risk to human health [5], whereas a low radiation dose could be insufficient to visualize anatomical structures. Therefore, it is important to perform radiological assessments using doses that are as low as clinically acceptable, without the loss of essential image quality [4].

Reconstructed images made by CBCT consist of voxels, which is the smallest image unit that determines the visibility of an image [6]. The size of each voxel is defined by its height, width, and thickness [7]. Voxel size is of principal importance in digitization time, reconstruction time, and condition of CBCT images [8, 9]. A smaller voxel size results in higher image resolution; however, higher irradiation is needed for a smaller voxel [10–12]. Moreover, the scanning time of objects becomes longer, which could increase the risk of patient movement [13]. Previous studies have suggested that images acquired in reasonable voxel sizes might provide acceptable diagnostic outcomes without increasing the risk of radiation exposure [8, 9, 14]. Diverse conclusions have been made on the voxel size that would be practicable. The use of voxel size smaller than 200 *μ*m has been suggested for detecting root fractures [15], while voxel size smaller than 160 *μ*m was suggested for the detection of root resorption [16]. In contrast, studies by Özer [17] and Liedke et al. [18] have reported that there were no significant differences among voxel sizes ranging from 125 *μ*m to 400 *μ*m for the detection of root fractures and root resorption in CBCT images.

In computer-guided implant surgery, an accurate three-dimensional (3D) image matching of hard and soft tissues obtained by CBCT and optical surface scanners is a prerequisite for prosthetic treatment planning, implant positioning, and surgical guide fabrication [19]. In the image registration process, when matching the CBCT and optical dental scan images, identical structures viewed in the two images are visually selected [20]. When the corresponding area appears different in shape, image matching could either be incomplete or not precise [21]. The final CBCT image depends on the operational parameters, and appropriate setting parameters that enable accurate image registration of intraoral optical scan to CBCT are still unclear. Thus, this study is aimed at assessing the effects of exposure parameters and voxel size for CBCT on image matching accuracy with an optical dental scan. The null hypothesis was that the accuracy of the image registration was not affected by exposure parameters and voxel size of CBCT.

## 2. Materials and Methods

### 2.1. Image Acquisition by Optical Dental Scan and CBCT

**Figure 1:**
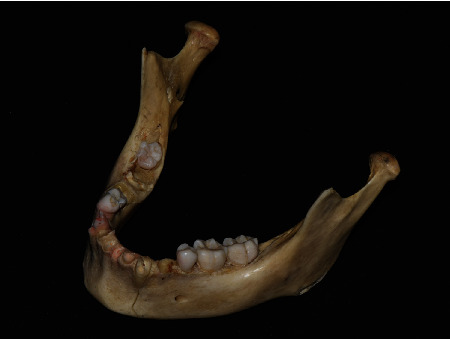
A dry human mandible without recent dental restoration.

A dry human mandible without recent dental restoration was included in the study (Figure 1). The surface data of the mandible was digitized using a lab-based scanner (IDC S1, Amann Girrbach, Kobach, Germany) and saved in the standard tessellation language (STL) format. Radiological data of the dry mandible were acquired in the digital imaging and communications in medicine (DICOM) format by using a dental CBCT scanner (PaX-i3D smart, Vatech, Hawseong, Korea). For this study, the FOV of 100 × 80 mm with a scanning time of 24 s was used for all scans, while the tube voltage was set at different values of 60, 80, and 100 kVp; the tube current was set at 6 and 8 mA; and the voxel size was set at 100, 200, and 300 *μ*m (a total of 18 experimental combinations). All CBCT scans were performed by an experienced radiology technician.

### 2.2. Image Registration of Optical Dental Scan to CBCT Image

**Figure 2:**
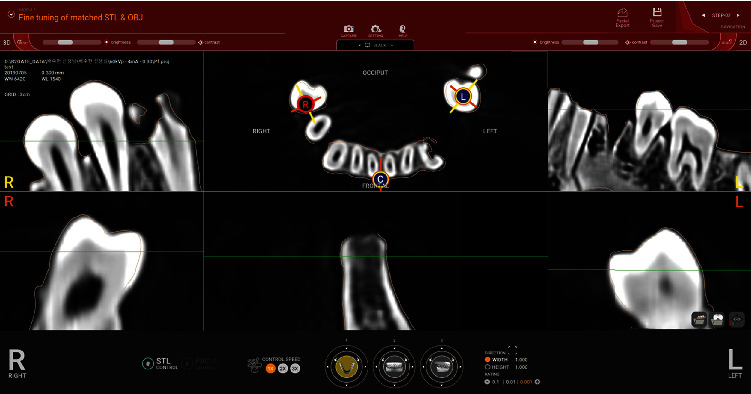
Image matching between cone-beam computed tomography and optical dental scan.

To merge the optical dental scan and the CBCT image, an image registration process was conducted in an implant planning software program (R2GATE v1.1.1, Megagen, Daegu, Korea). In each group, optical scan images were registered to the corresponding CBCT images based on the dental structures observable in both images, such as the incisal line angle or the cusp structure of the tooth. The optical scanned model was aligned to the 3D-reconstructed radiological model by the point-based best-fit algorithm. Thereafter, the optical scanned model was manually adjusted more to adapt the scan model to the radiological reconstructed data in 3D matching the individual representation of identical anatomical structures (Figure 2). Eighteen dental professionals experienced in using the implant planning software, who were blinded to the purpose of this study, performed the image registration process following instructions provided before the experimental phase.

### 2.3. Evaluation of the Accuracy of Image Registration

After the image registration step, DICOM data and optical scan were converted to 3D polygon models with the designated 3D orientations in the implant planning software and were transferred to an image analysis software program (Geomagic DesignX, 3D Systems, Rock Hill, SC, USA). The accuracy of image registration was analyzed by measuring the positional discrepancy between the registered optical scan image and the 3D-reconstructed radiological image. The linear discrepancy was assessed in the cross-sectional images of the remaining premolars using the “measure section” function of the software program. A single examiner, who had more than three years of experience in digital image analysis, carried out all measurements to minimize errors that can arise with different investigators.

### 2.4. Statistical Analysis

The mean and standard deviation of linear discrepancies in each CBCT condition were computed by averaging the measurement values assembled in the remaining tooth regions. A Kruskal-Wallis test and a post hoc Mann–Whitney *U* test with Bonferroni correction were used to nonparametrically compare the accuracy of image registration between the groups. Three-way analysis of variance (ANOVA) was used to investigate how interactions between CBCT conditions, including tube voltage, tube current, and voxel size, affected image registration accuracy. All statistical analyses were executed using the statistical package for the social sciences (SPSS) software program (SPSS version 25.0, IBM Inc., Armonk, NY, USA) with a significance level of 0.05.

## 3. Results

**Figure 3:**
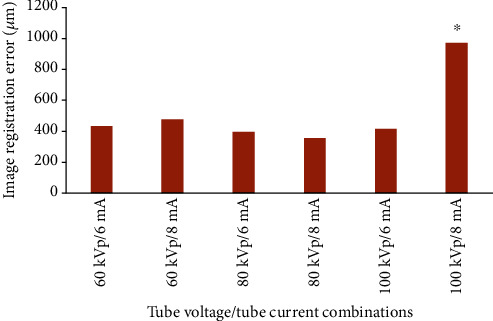
Image registration error between the optical scan and 3D-reconstructed radiological images derived from different combinations of tube voltage/tube current of cone-beam computed tomography. ^∗^Significant difference.

**Figure 4:**
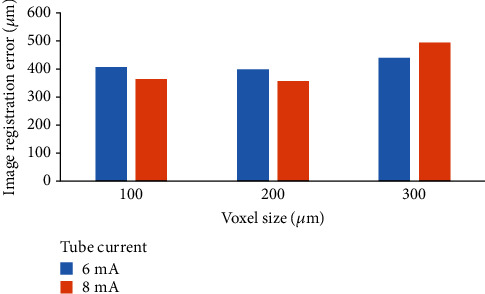
Image registration error between the optical scan and 3D-reconstructed radiological images derived from different voxel sizes of cone-beam computed tomography.

Table 1 shows the image registration error derived from different combinations of tube voltage, tube current, and voxel size for the experimental groups. The Kruskal-Wallis test revealed that the image registration accuracy was not significantly different between the voltage and current conditions; however, significantly higher image registration errors were found at the combination of 100 kVp tube voltage/8 mA tube current (*F* = 8.44, *P* < 0.001; Figure 3). Although higher image registration errors were observed at a voxel size of 300 *μ*m compared with a voxel size of 100 *μ*m and 200 *μ*m, the difference among groups was not significant (Figure 4).

No interaction was found among exposure parameters of tube voltage, tube current, and voxel size in terms of image registration accuracy (*F* = 2.022, *P* = 0.091). The combination of the tube voltage/tube current/voxel size at 80 kVp/8 mA/200 *μ*m showed the smallest image registration error (357.6 ± 146.1 *μ*m), while the highest error was found at 100 kVp/8 mA/200 *μ*m combination (970.9 ± 682.1 *μ*m).

## 4. Discussion

The purpose of this was to assess the effects of exposure parameters and voxel size for CBCT on the image matching accuracy between radiological and optical dental scan images. As regards the exposure conditions, high tube voltage/current could lower the image matching accuracy. The voxel size does not significantly affect the accuracy of image registration. No interaction was found among tube voltage, tube current, and voxel size in terms of the matching accuracy. Thus, the null hypothesis that the accuracy of the image registration was not affected by exposure parameters and voxel size of CBCT images was partially rejected.

In this study, the accuracy of image registration was not significantly different between the voltage and current conditions but was significantly low at the 100 kV and 8 mA combination. The effect of operation parameters on CBCT has been investigated by several researchers, with most reports focusing on the effect of radiation dose reduction on image quality [22, 23]. Diverse results have been reported, depending on the standard used to evaluate scan devices, image quality, amount of dose reduction, and exposure parameters. Most studies have found that a substantial dose reduction using exposure parameters below the manufacturer's guidelines is possible with adequate visibility [24–26]. Rivas et al. found that dose reductions to 50% lower than the manufacturer's default settings could be achieved by decreasing the X-ray tube voltage [27]. Practically, dose reduction cannot be achieved entirely by changing the tube voltage. Pauwels et al. stated that low-dose protocols should necessitate tube current reduction, rather than tube voltage reduction, because the increase in image artifact for a given dose reduction would be smaller [28]. Based on our study results, the highest accuracy of image registration was observed at 80 kVp/8 mA combination of exposure parameter setting for CBCT.

Reconstruction of a CBCT scan with a larger voxel size may produce images of lower spatial resolution [29]. Interestingly, based on the present results, no significant difference in the image matching accuracy was found among groups with a different voxel size of CBCT images. One possible reason is the effect of image noise. Although smaller voxel size images have more sharpness, the use of a smaller voxel size to increase the image resolution can create some noise [30]. By contrast, using large voxel size reduced image noise owing to averaging gray scales of photons through slices that caused less image noise [31]. Moreover, a large voxel size may not aggravate the distinguishability of anatomic structure images. Maret et al. examined the effect of voxel size on the accuracy of 3D reconstructions and volumetric changes in CBCT volumes and showed no difference in CBCT image with voxel size up to 200 *μ*m; the differences became significant starting from 300 *μ*m and above [32]. The voxel size of 300 *μ*m may not be the optimal condition for 3D reconstructions of CBCT, but acceptable for the image matching with optical scan images. Depending on the voxel size, some radiopaque structures can become invisible, and the volume reconstruction can become overestimated due to the partial volume effect [33, 34]. The partial volume effect occurs when a larger voxel does not lie completely within an object but lies at the border of two objects of different densities [33]. This voxel then would reflect an average density value of both objects rather than the true value of each object's density. These partial volume effects would bring artifacts that lead to unclear CBTC images [34]. Therefore, extra cautions should be taken while increasing the scanning voxel size because when the voxel size is too large, the risk of partial volume effect may be increased.

In this in vitro study, the use of the human dry skull without a soft tissue substitute may involve a drawback, as it does not simulate the actual anatomy in a real clinical situation. Dusseldorp et al. investigated the effect of soft tissue presence on the 3D image segmentation accuracy of hard tissue models from CBCT and concluded that, although the soft tissue presence appears to affect the accuracy of the 3D hard tissue model obtained from a CBCT scanner, discrepancies were below a generally clinical acceptable level of 1 mm [35]. Further studies should investigate how to optimize parameter settings to overcome the potential inaccuracy of image matching between CBCT and optical dental scan images in patient-based clinical trials.

## 5. Conclusion

Within the limits of this in vitro study, different exposure parameter settings in tube voltage and tube current did not influence the image matching accuracy; however, a high radiation dose can be avoided in a way that the imaging accuracy is maintained, by following the correct CBCT protocol. Generally, voxel sizes of CBCT did not significantly affect the image matching accuracy between CBCT and optical scan images.

## Figures and Tables

**Table 1:** Mean and standard deviation of image registration error between the optical scan and 3D-reconstructed radiological images derived from different combinations of tube voltage, tube current, and voxel size of cone-beam computed tomography.

Combination	Tube voltage (kVp)	Tube current (mA)	Voxel size (*μ*m)	Mean ± SD (*μ*m)
1	60		100	441.6 ± 296.0
2		6	200	436.7 ± 170.0
3			300	458.5 ± 179.8
4			100	407.6 ± 147.3
5		8	200	477.8 ± 258.3
6			300	488.9 ± 205.7
7	80		100	407.6 ± 143.1
8		6	200	399.9 ± 151.4
9			300	441.4 ± 220.5
10			100	365.5 ± 194.7
11		8	200	357.6 ± 146.1
12			300	494.2 ± 224.1
13	100		100	924.8 ± 260.9
14		6	200	414.0 ± 178.6
15			300	698.3 ± 319.6
16			100	530.0 ± 243.2
17		8	200	970.9 ± 682.1
18			300	454.2 ± 174.3

^∗^The field of view (FOV) of 100 × 80 mm with a scanning time of 24 s was used for all cone-beam computed tomography scans.

## Data Availability

The data used to support the findings of this study are included within the article.

## References

[1] Jacobs R., Salmon B., Codari M., Hassan B., Bornstein M. M. (2018). Cone beam computed tomography in implant dentistry: recommendations for clinical use. *BMC Oral Health*.

[2] Park H. N., Min C. K., Kim K. A., Koh K. J. (2019). Optimization of exposure parameters and relationship between subjective and technical image quality in cone-beam computed tomography. *Imaging Science in Dentistry*.

[3] Woo H. W., Mai H. N., Lee D. H. (2020). Comparison of the accuracy of image registration methods for merging optical scan and radiographic data in edentulous jaws. *Journal of Prosthodontics*.

[4] Bamba J., Araki K., Endo A., Okano T. (2013). Image quality assessment of three cone beam CT machines using the SEDENTEXCT CT phantom. *Dento Maxillo Facial Radiology*.

[5] Caruso S., Storti E., Nota A., Ehsani S., Gatto R. (2017). Temporomandibular joint anatomy assessed by CBCT images. *BioMed Research International*.

[6] Cho P. S., Johnson R. H., Griffin T. W. (1995). Cone-beam CT for radiotherapy applications. *Physics in Medicine & Biology*.

[7] Patcas R., Müller L., Ullrich O., Peltomäki T. (2012). Accuracy of cone-beam computed tomography at different resolutions assessed on the bony covering of the mandibular anterior teeth. *American Journal of Orthodontics and Dentofacial Orthopedics*.

[8] Dong T., Xia L., Cai C., Yuan L., Ye N., Fang B. (2019). Accuracy of in vitro mandibular volumetric measurements from CBCT of different voxel sizes with different segmentation threshold settings. *BMC Oral Health*.

[9] Dong T., Yuan L., Liu L. (2019). Detection of alveolar bone defects with three different voxel sizes of cone- beam computed tomography: an _in vitro_ study. *Scientific Reports*.

[10] Moshfeghi M., Tavakoli M. A., Hosseini E. T., Hosseini A. T., Hosseini I. T. (2012). Analysis of linear measurement accuracy obtained by cone beam computed tomography (CBCT-NewTom VG). *Dental Research Journal*.

[11] Farman A. G., Scarfe W. C. (2006). Development of imaging selection criteria and procedures should precede cephalometric assessment with cone-beam computed tomography. *American Journal of Orthodontics and Dentofacial Orthopedics*.

[12] Molen A. D. (2010). Considerations in the use of cone-beam computed tomography for buccal bone measurements. *American Journal of Orthodontics and Dentofacial Orthopedics*.

[13] Damstra J., Fourie Z., Slater J. J. H., Ren Y. (2010). Accuracy of linear measurements from cone-beam computed tomography-derived surface models of different voxel sizes. *American Journal of Orthodontics and Dentofacial Orthopedics*.

[14] Sang Y. H., Hu H. C., Lu S. H., Wu Y. W., Li W. R., Tang Z. H. (2016). Accuracy assessment of three-dimensional surface reconstructions of in vivo teeth from cone-beam computed tomography. *Chinese Medical Journal*.

[15] Melo S. L., Bortoluzzi E. A., Abreu Jr M., Corrêa L. R., Corrêa M. (2010). Diagnostic ability of a cone-beam computed tomography scan to assess longitudinal root fractures in prosthetically treated teeth. *Journal of Endodontics*.

[16] Kamburoğlu K., Kursun S. (2010). A comparison of the diagnostic accuracy of CBCT images of different voxel resolutions used to detect simulated small internal resorption cavities. *International Endodontic Journal*.

[17] Özer S. Y. (2011). Detection of vertical root fractures by using cone beam computed tomography with variable voxel sizes in an in vitro model. *Journal of Endodontics*.

[18] Liedke G. S., da Silveira H. E., da Silveira H. L., Dutra V., de Figueiredo J. A. (2009). Influence of voxel size in the diagnostic ability of cone beam tomography to evaluate simulated external root resorption. *Journal of Endodontics*.

[19] Mai H. N., Lee D. H. (2021). Radiopaque tissue surface-based digital registration technique for completely edentulous ridge. *The Journal of Oral Implantology*.

[20] Al-Rimawi A., Shaheen E., Albdour E. A., Shujaat S., Politis C., Jacobs R. (2019). Trueness of cone beam computed tomography versus intra-oral scanner derived three-dimensional digital models: an ex vivo study. *Clinical Oral Implants Research*.

[21] Nkenke E., Zachow S., Benz M. (2004). Fusion of computed tomography data and optical 3D images of the dentition for streak artefact correction in the simulation of orthognathic surgery. *Dento Maxillo Facial Radiology*.

[22] Goulston R., Davies J., Horner K., Murphy F. (2016). Dose optimization by altering the operating potential and tube current exposure time product in dental cone beam CT: a systematic review. *Dento Maxillo Facial Radiology*.

[23] Costa A. L. F., Barbosa J.-P. P.-G., Perez-Gomes J. P., Calle A. J. M., Santamaria M. P., Lopes S. L. P. C. (2018). Influence of voxel size on the accuracy of linear measurements of the condyle in images of cone beam computed tomography: a pilot study. *Journal of Clinical and Experimental Dentistry*.

[24] Kwong J. C., Palomo J. M., Landers M. A., Figueroa A., Hans M. G. (2008). Image quality produced by different cone-beam computed tomography settings. *American Journal of Orthodontics and Dentofacial Orthopedics*.

[25] Dawood A., Brown J., Sauret-Jackson V., Purkayastha S. (2012). Optimization of cone beam CT exposure for pre-surgical evaluation of the implant site. *Dento Maxillo Facial Radiology*.

[26] Thanasupsombat C., Thongvigitmanee S. S., Aootaphao S., Thajchayapong P. (2018). A simple scatter reduction method in cone-beam computed tomography for dental and maxillofacial applications based on Monte Carlo simulation. *BioMed Research International*.

[27] Hidalgo Rivas J. A., Horner K., Thiruvenkatachari B., Davies J., Theodorakou C. (2015). Development of a low-dose protocol for cone beam CT examinations of the anterior maxilla in children. *The British Journal of Radiology*.

[28] Pauwels R., Silkosessak O., Jacobs R., Bogaerts R., Bosmans H., Panmekiate S. (2014). A pragmatic approach to determine the optimal kVp in cone beam CT: balancing contrast-to-noise ratio and radiation dose. *Dento Maxillo Facial Radiology*.

[29] Eliliwi M., Bazina M., Palomo J. M. (2020). kVp, mA, and voxel size effect on 3D voxel-based superimposition. *The Angle Orthodontist*.

[30] Tanimoto H., Arai Y. (2009). The effect of voxel size on image reconstruction in cone-beam computed tomography. *Oral Radiology*.

[31] Hassan B., Couto Souza P., Jacobs R., de Azambuja Berti S., van der Stelt P. (2010). Influence of scanning and reconstruction parameters on quality of three-dimensional surface models of the dental arches from cone beam computed tomography. *Clinical Oral Investigations*.

[32] Maret D., Telmon N., Peters O. A. (2012). Effect of voxel size on the accuracy of 3D reconstructions with cone beam CT. *Dento Maxillo Facial Radiology*.

[33] Spin-Neto R., Gotfredsen E., Wenzel A. (2013). Impact of voxel size variation on CBCT-based diagnostic outcome in dentistry: a systematic review. *Journal of Digital Imaging*.

[34] Ye N., Jian F., Lai W. (2013). Effect of voxel size and partial volume effect on accuracy of tooth volumetric measurements with cone beam CT. *Dento Maxillo Facial Radiology*.

[35] Dusseldorp J., Stamatakis H., Ren Y. (2017). Soft tissue coverage on the segmentation accuracy of the 3D surface-rendered model from cone-beam CT. *Clinical Oral Investigations*.

